# Production of Nanoemulsions from Palm-Based Tocotrienol Rich Fraction by Microfluidization

**DOI:** 10.3390/molecules201119666

**Published:** 2015-11-05

**Authors:** Pik Seah Goh, Mei Han Ng, Yuen May Choo, Amru Nasrulhaq Boyce, Cheng Hock Chuah

**Affiliations:** 1 Institute of Biological Sciences, Faculty of Science, University of Malaya, 50603 Kuala Lumpur, Malaysia; selcygoh@hotmail.com (P.S.G.); amru@um.edu.my (N.B.A.); 2 Milling & Processing Unit, Engineering & Processing Division, Malaysian Palm Oil Board, 6 Persiaran Institusi, Bandar Baru Bangi, 43000 Kajang, Malaysia; choo@mpob.gov.my; 3 Department of Chemistry, Faculty of Science, University of Malaya, 50603 Kuala Lumpur, Malaysia

**Keywords:** nanoemulsion, microfluidization, tocotrienols, nonionic emulsifiers

## Abstract

In the present study, tocotrienol rich fraction (TRF) nanoemulsions were produced as an alternative approach to improve solubility and absorption of tocotrienols. In the present study, droplet size obtained after 10 cycles of homogenization with increasing pressure was found to decrease from 120 to 65.1 nm. Nanoemulsions stabilized with Tween series alone or emulsifier blend Brij 35:Span 80 (0.6:0.4 *w*/*w*) homogenized at 25,000 psi and 10 cycles, produced droplet size less than 100 nm and a narrow size distribution with a polydispersity index (PDI) value lower than 0.2. However blend of Tween series with Span 80 produced nanoemulsions with droplet size larger than 200 nm. This work has also demonstrated the amount of tocols losses in TRF nanoemulsion stabilized Tweens alone or emulsifier blend Brij 35:Span 80 (0.6:0.4 *w*/*w*) ranged between 3%–25%. This can be attributed to the interfacial film formed surrounding the droplets exhibited different level of oxidative stability against heat and free radicals created during high pressure emulsification.

## 1. Introduction

Palm oil is well known for its abundant natural source of vitamin E, carotenoids and polyunsaturated fatty acids [1]. These functional lipids have gained increasing interest for their applications in the food, pharmaceutical and cosmetics industries due to their enormous health benefits. Vitamin E comprise of two main groups: tocopherols and tocotrienols. Both groups share similar chemical structure which contains a chromane ring and an alkyl side chain. However, tocopherol has a saturated side chain whereas tocotrienol has three double bonds at positions 3′, 7′, and 11′ at the alkyl side chain [2]. Another group known as tocomonoenol with a double bond at the position 11′ of the alkyl side chain was reported in 2004 [2]. Both tocopherols and tocotrienols contain four derivatives: alpha (α-), beta (β-), gamma (γ-) and delta (δ-), that differ from each other based on the number and position of methyl groups attached to their chromane rings [3]. It is well documented that tocopherols and tocotrienols play an essential role in preventing numerous degenerative diseases such as cancer, atherosclerosis, neurodegeneration and aging due to oxidation caused by reactive oxygen and nitrogen species [1,4]. Some researchers believe that tocotrienols exert more potent anticancer activity compared to tocopherols or its derivatives [5]. Although they exhibit high nutritional benefits for human, absorption of vitamin E through oral administration has been reportedly ineffective due to its poor aqueous solubility and low intestinal permeability. Furthermore, they are highly susceptible to oxidation when exposed to heat, light or oxygen. Therefore, an efficient delivery system to overcome low solubility and intestinal permeability as well as to improve the stability of vitamin E from degradation during storage or processing is essential.

In recent years, nano-scale delivery systems, such as in the form of nanoemulsion, has become a promising approach for improving the solubility and intestinal permeability of vitamin E [6,7]. They are known as nanoemulsion or submicron emulsion with their average oil droplet diameter in the range of 10–100 nm. It appears optically transparent or slightly turbid since the oil droplets diameter are relatively smaller compared to the wavelength of light (λ = 390–750 nm) and thus scatter light weakly [8]. The optical appearance of nanoemulsion enabled it to be utilized by incorporating poor water soluble compounds, such as vitamins, flavors and drugs into transparent aqueous-based food and beverage [8]. Nanoemulsion is considered to be kinetically stable as it exhibits random Brownian motion that is sufficient in overcoming gravitational separation and thus prevents emulsion droplets from creaming and sedimentating as well as re-coalescence and flocculation. Alqahtani *et al.* [9] demonstrated that tocotrienols delivered in the form of nanoemulsion can successfully improve oral bioavailability of tocotrienols by enhancing passive permeability across the intestinal membrane.

Nanoemulsion can be fabricated by low energy or high energy emulsification approaches. High energy emulsification requires the input of energy to generate disruptive forces that mechanically breakup large oil droplets to form fine droplets that are suspended within the aqueous phase [8,10]. One of the advantages of using this approach is that nanoemulsion can be easily achieved by using a lower amount of emulsifier as compared to when they are required for incorporation into the emulsion system generated by low energy emulsification [11]. In addition, it offers flexible control over droplet size and droplet size distribution and a greater improvement in stability, as well as being potentially applicable for large-scale industrial production. Energy-intensive technologies such as ultrasonication, high pressure homogenization and microfluidization are commonly used for the preparation of nanoemulsions [11]. Some researchers have suggested that emulsification efficiency in a microfluidization is superior compared to ultrasonication and high pressure homogenization, as it is able to generate emulsion with smaller droplet size and narrower droplet size distribution [12,13].

Food grade emulsifiers are highly recommended in producing edible nanoemulsions. They comprise of large molecular weight emulsifiers such as whey protein isolate (WPI), whey protein concentrate (WPC), soy protein isolate (SPI) and gum Arabic. However their stabilization of newly formed interfaces (droplets) are poor due to slow diffusion and absorption rate [11,14]. Small molecular weight emulsifiers, such as a nonionic emulsifier has been widely used in stabilizing oil in water emulsions. They are very mobile and can absorbed quickly onto a fresh interface to prevent re-coalescence through steric repulsion. In addition, nonionic emulsifier exhibits low oral toxicity and causes less irritation to cellular surfaces and can be considered as the emulsifier of choice in stabilizing nanoemulsions [15].

In this study, we examined the effect of pressure in microfluidizer homogenization and number of cycles in order to produce tocotrienol rich fraction (TRF) nanoemulsions with droplet size smaller than 100 nm and a narrow droplet size distribution. The effects of different types of emulsifiers on the physicochemical properties of the resulting TRF nanoemulsions were also investigated.

## 2. Results and Discussion

### 2.1. Effect of Microfluidization Conditions on Droplet Size and PDI of TRF Nanoemulsions

**Figure 1:**
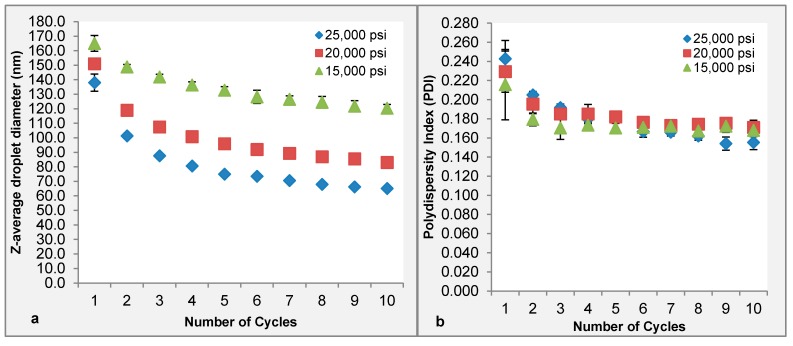
Influence of operating pressure and number of cycles on mean droplet diameter (**a**) and polydispersity index (PDI) (**b**) of tocotrienol rich fraction (TRF) nanoemulsions prepared with emulsifier blend Span 80:Brij 35 (0.4:0.6 *w*/*w*) at a concentration of 2.5% *w*/*v*.

**Figure 2:**
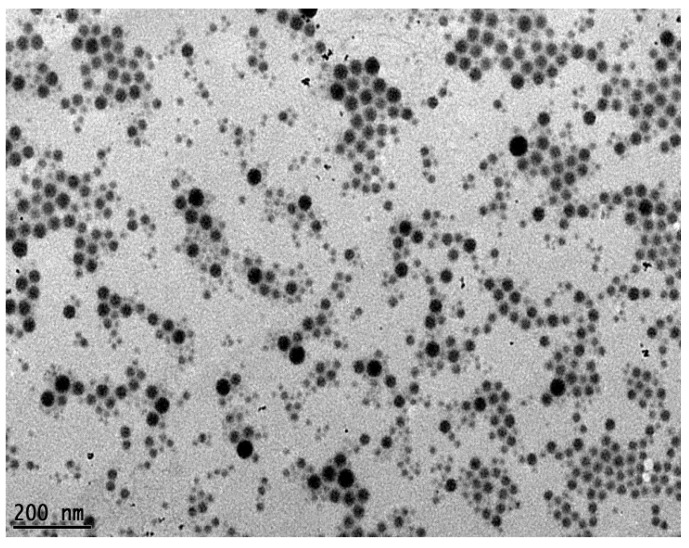
Spherical oil droplets of TRF nanoemulsion prepared with emulsifier blend Span 80:Brij 35 (0.4:0.6 *w*/*w*) at a concentration of 2.5% *w*/*v* homogenized by microfluidizer (25,000 psi, 10 passes) viewed under transmission electron microscopy (TEM).

The present results showed that increasing homogenization pressure promotes droplets deformation followed by disruption leading to reduction of oil droplet size (Figure 1a). The mean droplet diameter of TRF nanoemulsions decreased significantly (*p* < 0.05) with increasing homogenization pressure, which were in agreement with findings of previous literature [11,14]. When the emulsion was homogenized at 15,000 psi, 20,000 psi and 25,000 psi for three cycles, it was observed that the average droplet size reduced gradually from 141.8 ± 1.9 to 107.4 ± 1.7 and to 87.6 ± 0.8 nm respectively. This indicated that larger droplets that were not disrupted at lower homogenization pressure were disrupted as the pressure increased due to the higher magnitude of shearing forces generated within the interaction chamber [11,16]. Closer observation revealed that all the emulsions generated at the three different pressures showed initial reduction in droplet size and a leveling-off period after seven cycles whereby no greater reduction in average droplet size was observed. Similar result was also observed on the polydispersity index (PDI) of emulsions with the increasing number of homogenization cycle (Figure 1b). After 10 homogenization cycles, the PDI values obtained reduced from 0.243 ± 0.019 to 0.155 ± 0.008 for 25,000 psi, 0.229 ± 0.022 to 0.171 ± 0.008 for 20,000 psi and 0.216 ± 0.037 to 0.168 ± 0.008 for 15,000 psi respectively, indicating emulsions with narrower size distribution were produced. Previous findings reported that during homogenization, droplets nearest to the walls of the channels experienced lower shearing force compared to the droplets away from the walls, leaving larger oil droplets undisrupted [16]. In a nutshell, the present study showed that subjecting the emulsions through an interaction chamber for a few cycles and at higher homogenization pressure ensures disruption of larger droplets to produce emulsions with a more uniform size (PDI values lower than 0.2). Results showed that a homogenization pressure of 25,000 psi for 10 cycles produced the smallest particle. 25,000 psi is thus used in subsequent experiments to study the effect of emulsifier type on the physical and chemical properties of TRF nanoemulsions. Based on TEM observations, no large oil droplets were observed and they were spherical in shape and uniform in size (Figure 2).

### 2.2. Effect of Different Emulsifiers on the Physical Properties of TRF Nanoemulsions and Tocols Content

Span 80 is a lipophilic emulsifying liquid agent which tends to form water-in-oil emulsions. Tween series is a derivative of Span whereby the hydroxyl groups on the sorbitan ring are substituted with polyoxyethylene groups. Tween series is more water soluble and widely used as stabilizing agent in oil-in-water emulsion formation. Brij 35 is a water soluble emulsifier comprising a hydrophilic chain that contains 23 ethoxylate groups. Both Tween series and Brij 35 have identical tails comprising methylene groups. The mixed hydrophilic-lipophilic balance (HLB) values were calculated using the following equation established by Griffin (1949) [17]:

(1) HLB*_mix_* = HLB*_T_* T% + HLB*_S_* S%

where HLB*_T_*, HLB*_S_* and HLB*_mix_* are the HLB values of Tween series or Brij 35, Span 80 and the mixed surfactants respectively. T% is the mass percentages of Tween series or Brij 35 and S% is the mass percentages of Span 80 in the mixed surfactants, respectively. Initial HLB values for Tween series, Brij 35 and Span 80 was adapted from manufacturer’s specification.

As can be seen in Table 1, nanoemulsions stabilized by one emulsifier alone had an average droplet size less than 100 nm, whereas average droplet size for emulsions stabilized by a blend of two emulsifiers was greater than 200 nm, with the exception of the Span 80/Brij 35 mixture which produced an average droplet size of 66.0 ± 1.9 nm. Present results also showed that although the average droplet size obtained was lowest (44.0 ± 1.0 nm) in emulsions stabilized by Brij 35, the emulsions were highly polydispersed (PDI = 0.561), with bimodal distribution observed indicating the presence of a mixture of large and small TRF droplets. Polydispersed emulsions are considered unstable and tend to breakdown over time due to the Ostwald ripening process which leads to emulsion coarsening [18]. It is noteworthy that in the present study, the addition of Span 80 improved size distribution by obtaining a lower PDI. This can be attributed to the emulsifier blend of Span 80 and Brij 35 besides able to reduce the interfacial tension and facilitate disruption of larger oil droplets, the interfacial film elasticity that form surrounding the oil droplets may have play a role in preventing droplets coalescence by possessing enough elasticity to remain intact and not easily rupture when droplets are compressed against each other. However, investigation on the interfacial tension and interfacial dilational elasticity formed by emulsifier blend of Span 80 and Brij 35 is suggested to be carried out in future study in order to provide a better understanding on the interface formed.

**Table 1:** Z-average diameter (nm), PDI and ζ-potential (mV) of TRF nanoemulsions prepared with different emulsifiers (mean ± S.D., *n* = 3).

Emulsifier	HLB Value	Z-Average Diameter (nm) *	PDI *	ζ-Potential (mV) *
Tween 20	16.7	55.9 ± 0.8 ^d,e^	0.154 ± 0.007 ^c^	−37.2 ± 3.4 ^a,b^
Tween 40	15.6	72.2 ± 0.8 ^b,c,d^	0.169 ± 0.003 ^c^	−32.5 ± 1.1 ^a^
Tween 60	14.9	90.8 ± 4.2 ^b^	0.185 ± 0.011 ^b,c^	−30.7 ± 0.7 ^a^
Tween 80	15.0	78.3 ± 1.5 ^b,c^	0.163 ± 0.0 ^c^	−39.1 ± 1.6 ^a,b^
Brij 35	16.9	44.0 ± 1.0 ^e^	0.561 ± 0.037 ^a^	−42.8 ± 2.4 ^b,c^
Span 80:Tween 20 (0.4:0.6 *w*/*w*)	11.74	192.9 ± 8.6 ^a^	0.232 ± 0.025 ^b^	−54.6 ± 1.1 ^d^
Span 80:Tween 40 (0.4:0.6 *w*/*w*)	11.08	177.0 ± 1.8 ^a^	0.199 ± 0.003 ^b,c^	−51.2 ± 7.7 ^c,d^
Span 80:Tween 60 (0.4:0.6 *w*/*w*)	10.66	189.3 ± 9.4 ^a^	0.198 ± 0.011 ^b,c^	−51.5 ± 2.6 ^c,d^
Span 80:Tween 80 (0.4:0.6 *w*/*w*)	10.72	195.3 ± 18.8 ^a^	0.231 ± 0.010 ^b^	−58.3 ± 4.2 ^d^
Span 80:Brij 35 (0.4:0.6 *w*/*w*)	11.86	66.0 ± 1.9 ^c,d^	0.173 ± 0.029 ^c^	−39.9 ± 2.1 ^a,b^

* Means within each column with different letters are significantly different from each other (*p* < 0.05).

It was observed that an emulsion stabilized by blend of Span 80 with different Tweens did not provide a synergistic effect in facilitating droplet deformation. The average droplets diameter from the resulting nanoemulsion ranged between 177.0 ± 1.8 and 195.3 ± 18.8 nm. This indicated that the mixture of Span and Tween was not effective in reducing the interfacial tension of the oil/water interface that facilitates droplets disruption. It has been suggested that emulsifiers with higher hydrophilic-lipophilic balance (HLB) values may play a role in producing smaller droplet diameter as greater hydrophilicity can stabilize the particles in an O/W emulsion more efficiently and absorb more rapidly on newly formed droplet surface during homogenization [19].

Although non-ionic emulsifiers do not provide electrical charge to oil droplet surface, the negative zeta potential (ζ-potential) value observed may be due to the adsorption of OH^−^ ions from the aqueous phase or cationic impurities from the droplet interface and presence of anionic hydrocolloid sodium alginate and gum Arabic that adsorbs to the interfacial layer [14,16]. However, as can be seen in Table 1, emulsifiers that produced emulsion droplet size <100 nm had similar ζ-potential values which ranged between −42.8 ± 2.4 and −30.7 ± 0.7. Whereas, emulsion with droplet size >100 nm (stabilized by blend of Span 80 and different Tweens) had higher negative ζ-potential values. Previous studies by Berton *et al.* [20] and Lu and Rhodes [21] reported that Span/Tween blends exhibited a strong repulsive interaction which led to inhomogeneous distribution of surfactants at the oil-water interfaces, forming a loosely packed interfacial film. In present study, during high pressure homogenization the newly formed oil droplets surface was loosely covered by the Span 80/Tweens mixtures due to them repelling each other. This increases the tendency for droplet re-coalescence. As compared to the Span 80/Tweens mixture, emulsions stabilized by a Span 80/Brij 35 blend exhibited weaker repulsive forces (lower negative ζ-potential value = −39.9). This promoted a more homogenous distribution of Span 80 in the film of Brij 35 surrounding the TRF droplets. The ζ-potential values obtained in the present study could be an indicator that there was a strong repulsive interaction between Span 80 and different Tweens.

As mentioned in Tan and Nakajima [22], during reduction of droplet size degradation of these oil soluble bioactive compounds it is inevitable due to increasing surface area of the droplets leading to increased exposure to light and free radicals that might have been created from the rise of temperature under high pressure shearing activity. As such tocols content of TRF nanoemulsion with droplet size smaller than 100 nm was analyzed to determine the amount of losses. Overall the percentage of tocols degraded during processing varied from 3.9% to 25.1% and was significantly (*p* < 0.05) influenced by the type of emulsifiers used (Table 2). Approximate 10% of the tocols degraded in TRF nanoemulsions stabilized by Tweens alone. Whereas, TRF nanoemulsion stabilized by mixture of Span 80:Brij 35 suffers a greater loss whereby a quarter of the tocols there degraded during processing. This observation can be attributed to the interfacial film formed by emulsifiers that served as a protective barrier for tocols that displayed different levels of oxidative stability against free radicals created during processing [19,20].

**Table 2:** Concentration of tocols in TRF nanoemulsion prepared with different emulsifiers (mean ± S.D., *n* = 3).

Emulsifier	Before Microfluidization (mg/g) *	After Microfluidization (mg/g)	Percentage Loss of Tocols (%) **
Tween 20	730.7 ± 41.5	653.3 ± 11.0	10.5 ± 3.7 ^a^
Tween 40	712.8 ± 39.7	684.7 ± 34.0	3.9 ± 1.0 ^a^
Tween 60	727.5 ± 3.5	675.3 ± 17.1	7.2 ± 2.4 ^a^
Tween 80	811.7 ± 9.7	777.6 ± 19.7	4.2 ± 1.3 ^a^
Brij 35	737.0 ± 60.6	677.1 ± 73.8	8.2 ± 2.9 ^a^
Span 80:Brij 35 (0.4:0.6 *w*/*w*)	708.3 ± 44.5	530.0 ± 23.1	25.1 ± 2.7 ^b^

* mg of tocols per g of tocotrienols rich fraction (TRF) palm oil; ** Means within each column with different letters are significantly different from each other ( *p* < 0.05).

Nevertheless, it was observed that even though TRF nanoemulsion was emulsified under high pressure system (25,000 psi), the percentage of tocol loss was less than 30%. This could occur during microfluidization, as the duration required for emulsion entering and exiting the interaction chamber into the cooling coil occurred in split seconds. Therefore, this helps to minimize the losses in tocols when exposed to the heat dissipated during high pressure homogenization. The percentage of losses recorded in this study were also lower compared to previous findings [23,24]. This can be attributed to the omission of an evaporation process to remove the organic solvent used during nanoemulsions preparation as reported in previous literature [23,24]. The results have shown the potential of microfluidization technique to produce nanoemulsions under organic solvent free conditions which is applicable in food or pharmaceutical industries.

### 2.3. Physical Stability of TRF Nanoemulsions during Storage

The stability of TRF nanoemulsions produced was studied over a month at 25 °C and 4 °C. The nanoemulsions were stabilized either by using Tween 20 or a combination of Span 80:Brij 35 (0.4:0.6 *w*/*w*) as both produced similar average droplet size. As shown in Table 3, the results demonstrated that average droplet size, PDI and ζ-potential remain fairly unchanged throughout the storage period regardless of storage temperature. TRF nanoemulsions stabilized by either Tween 20 or the Span 80:Brij 35 blend, exhibited great stability before and after storage. To date, mostly Vitamin E nanoemulsion was formed using Tweens; limited studies have used Brij 35 to stabilize Vitamin E nanoemulsion. Laouini *et al.* [25] had reported that they used of blend Tween 80:Brij 35 which enabled them to produce nanoemulsion with droplet size less than 100 nm. In the present study, a stable TRF nanoemulsion with droplet size less than 100 nm and a narrow PDI was achieved using emulsifier blend of Span 80:Brij 35. The formed TRF nanoemulsions will be used to produce microparticle through spray drying. Hence, nanoemulsions that possess high stability is highly favorable in order to produce microparticles that exhibit high encapsulation efficiency [26].

## 3. Experimental Section

### 3.1. Materials

Tocotrienol Rich Fraction (TRF) (labelled as Super T75) derived from palm oil containing a mixture of 16.8% α-tocopherol, 19.1% α-tocotrienol. 1.8% β-tocotrienol, 26.2% γ-tocotrienol and 11.9% δ-tocotrienol was purchased from SuperVitamins Sdn. Bhd, Johor, Malaysia. Sodium alginate, polyoxyethylene sorbitan monolaurate (Tween 20), polyoxyethylene sorbitan monopalmitate (Tween 40), polyoxyethylene sorbitan monostearate (Tween 60) and polyoxyethylene sorbitan monooleate (Tween 80) were purchased from R & M chemicals, Essex, UK. Gum Arabic, sorbitan monooleate (Span 80), polyoxyethylene lauryl ether (Brij 35), hexane and heptane were purchased from Merck, Darmstadt, Germany. All chemicals and reagents used were of analytical grade. Distilled water was used for all emulsion preparation.

### 3.2. Preparation of TRF Nanoemulsions

Sodium alginate (1% *w*/*v*) and gum Arabic (10% *w*/*v*) aqueous solutions were prepared separately using distilled water up to a volume of 100 mL each. Sodium alginate was dissolved in distilled water heated at 70 °C and the solution was left to cool to room temperature. Gum Arabic was dissolved in distilled water at room temperature. The alginate solution was mixed in equal quantity with the gum arabic solution to obtain an alginate (0.5% *w*/*v*)/gum arabic (5% *w*/*v*) mixture. Emulsifiers (2.5% *w*/*v*) were added into the mixture. Emulsifier concentration over TRF concentration used throughout the study was maintained at 1:1 *w*/*w* ratio. The mixture solution containing emulsifier was later added into a 250 mL beaker that contained weighed amount of TRF (2.5% *w*/*v*). The nanoemulsion was produced by two stage homogenization. The mixture was mixed using an overhead stirrer (IKA RW 20 digital, Staufen, Germany) at 8000 rpm for 5 min to produce a coarse primary emulsion. The resulting coarse emulsion was homogenized further by passing through a benchtop microfluidizer M-110P equipped with an interaction chamber F20Y, 75 µm (Microfluidics, Westwood, MA, USA).

**Table 3:** Stability of nanoemulsion prepared by Tween 20 and emulsifier combination Span 80:Brij 35 stored under temperature of 4 °C and 25 °C respectively (mean ± S.D., *n* = 3).

Storage Duration (Days)	0 *	7		14		21		28	
**(a) Emulsion prepared by Tween 20:**									
Storage temperature (°C)	25	4	25	4	25	4	25	4	25
Z-average diameter (nm)	72.5 ± 1.3	70.7 ± 1.8	73.4 ± 1.3	71.1 ± 1.6	73.6 ± 1.1	69.2 ± 1.8	72.8 ± 0.7	70.2 ± 1.3	73.9 ± 0.2
PDI	0.146 ± 0.006	0.151 ± 0.003	0.144 ± 0.003	0.151 ± 0.007	0.140 ± 0.005	0.151 ± 0.000	0.134 ± 0.006	0.149 ± 0.003	0.133 ± 0.009
ζ-potential (mV)	−43.6 ± 1.4	−43.2 ± 0.9	−44.9 ± 1.0	−43.3 ± 2.8	−43.5 ± 2.2	−42.2 ± 1.0	−42.6 ±0.9	−41.6 ± 0.6	−41.9 ± 0.5
**(b) Emulsion prepared by Span 80:Brij 35 (0.4:0.6 *w*/*w*)**									
Z-average diameter (nm)	66.2 ± 0.5	62.8 ± 0.7	66.2 ± 0.4	62.4 ± 0.8	66.0 ± 0.3	62.4 ± 1.1	65.8 ± 0.7	63.2 ± 1.2	66.2 ± 0.5
PDI	0.144 ± 0.007	0.162 ± 0.005	0.149 ± 0.003	0.162 ± 0.003	0.157 ± 0.005	0.162 ± 0.006	0.152 ± 0.002	0.160 ± 0.008	0.151 ± 0.005
ζ-potential (mV)	−35.9 ± 0.4	−43.2 ± 2.2	−45.0 ± 0.8	−43.1 ± 0.6	−45.6 ± 1.1	−42.7 ± 1.0	−44.8 ± 1.0	−43.0 ± 1.7	−44.9 ± 0.5

* One day zero, the Z-average diameter (nm) and PDI was only reported at room temperature (25 °C). As soon as the nanoemulsion was prepared, analysis of droplet size and PDI was carried out on the same day.

Emulsification by way of microfluidization was carried out at various pressure (15,000–25,000 psi) and number of homogenization cycles (1–10 cycles). At each operating pressure, emulsions were passed through the emulsification chamber and were taken for analysis after each cycle up to 10 cycles. As soon as the emulsion exits the interaction chamber, it enters an open coiled-type cooling jacket or heat exchanger for heat removal in order to cool the nanoemulsion formed to room temperature (25 °C). Samples were prepared in triplicate throughout the study. For storage tests, nanoemulsions were stored in screw-capped bottles covered with aluminium foil at 4 °C and 25 °C for 4 weeks. At the end of each week, the emulsions were sampled to determine its particle size.

### 3.3. Determination of Droplet Diameter, Polydispersity Index (PDI) and Zeta Potential (ζ-Potential)

Oil droplet diameter, PDI and ζ-potential of nanoemulsion were measured using dynamic light scattering Zetasizer Nano-ZS (Malvern Instruments, Worcestershire, UK). The emulsions were diluted with distilled water in a ratio (1:100 *v*/*v*) prior to analysis to avoid the effect of multiple scattering [27,28]. Cumulant analysis was used to determine the intensity-weighted mean diameter (Z-average) and polydispersity index (PDI) which indicated the broadness of the size distribution. Smoluchowski equation was employed to calculate the ζ-potential values from the measured velocity. Results are reported as average measurements from three freshly prepared samples.

### 3.4. Analysis of Tocols (Tocotrienol-Tocopherol) Content

**Figure 3:**
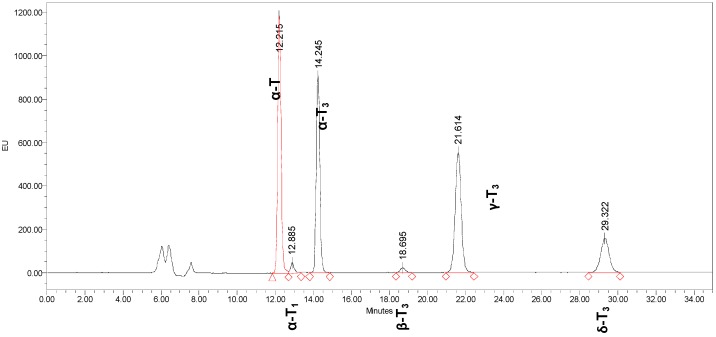
High performance liquid chromatography (HPLC) profile of vitamin E in palm TRF. Separation by Luna 5μ Silica (Phenomenex, Torrance, CA, USA) with normal phase heptane:ethyl acetate (94:6 *v*/*v*). α-T, α-tocopherol; α-T1, α-tocomonoenol; α-T3, α-tocotrienol; γ-T3, γ-tocotrienol; δ-T3, δ-tocotrienol.

The sample preparation procedure was modified from Laouini *et al.* [25]. 2 mL distilled water was added to 0.5 g emulsion to dilute the emulsion. The diluted emulsion was transferred into a 15 mL centrifuge tube. Hexane was added for extraction and after agitation for 2 min, the hexane layer was separated by centrifugation at 5000 rpm for 10 min. The upper hexane layer was collected. The extraction procedure was repeated twice. The collected hexane was purged to dryness by stream of nitrogen (N_2_). The oil extracted from the emulsion sample was weighed and re-dissolved in heptane for high performance liquid chromatography (HPLC) analysis using Waters 600 Controller equipped with Waters 2475 multi λ Fluorescence Detector (Waters, USA). Column Luna 5µ Silica 4.6 mm i.d. × 250 mm length purchased from Phenomenax (Torrance, CA, USA). The mobile phase consists of heptane and ethyl acetate (94:6 *v*/*v*) at the flow rate 0.6 mL/min. Detection was performed at excitation 295 nm and emission 325 nm. HPLC profile of palm TRF was presented in Figure 3. Calibration was carried out using an external standard comprised of: α-tocopherol 8.94. ppm, α-tocomonoenol 0.54ppm, α-tocotrienol 8.4 ppm, β-tocotrienol 0.84 ppm, γ-tocotrienol 10.5 ppm and δ-tocotrienol 3.66 ppm. Quantification of tocols was calculated based on the following formula obtained from Dauqan *et al*. [29] and was reported as mg of tocols/g of TRF:

(2) Tocols concentration (ppm) = As/Astd × Vs/Ws × Cstd × VIstd/Vis

where As = Area of sample; Astd = Area of standard; Vs = Volume of sample; Ws = Weight of sample; Cstd = Concentration of standard; VIstd = Volume of standard injected; Vis = Volume of sample injected.

### 3.5. Transmission Electron Microscopy (HR-TEM)

Transmission electron microscopy (JEOL-JEMP-2100F, Tokyo, Akishima, Japan) was used to investigate the morphology of nanoemulsions prepared by the microfluidizer. The preparation method was modified from Tang *et al*. [30]. The emulsion was diluted with distilled water followed by staining with 2% osmium oxide for an hour. A drop of nanoemulsion was added onto 200-mesh formwar carbon-coated copper grid (Agar Scientific Ltd., Essex, UK) and was left standing for 30 min. Excess solution was later drawn-off with a Whatman filter paper and left to dry at room temperature before analysis under TEM.

### 3.6. Statistical Analysis

All samples were prepared in triplicate. Results from all measurements were reported as means and standard deviations. The physicochemical properties of the palm-based TRF nanoemulsion were subjected to one-way analysis of variance (ANOVA) using R, an open source statistical software program [31]. Significant differences between means were determined by Tukey’s post hoc test with type of emulsifiers used for preparation as the fixed effect.

## 4. Conclusions 

The present study has shown that microfluidization using high shearing forces is a feasible methodology to obtain nano-size palm-based TRF emulsion, through manipulation of homogenization pressure and number of cycles. TRF nanoemulsions with average droplet size lower than 100 nm were obtained under 25,000 psi after 10 cycles. Results also demonstrated that the type of emulsifiers used had a significant effect on the physicochemical properties of the resulting TRF nanoemulsions. Combining Span 80 with different types of Tweens did not provide any synergistic effect in producing smaller droplet size, which could have mainly been due to the repulsive interaction between Span and Tween that led to the formation of a loosely packed interfacial film. Furthermore, stability of tocols against degradation might also be affected by the emulsifiers used as the percentage of tocols losses in TRF nanoemulsion, stabilized by Tween (10%), are lower compared to TRF nanoemulsion stabilized by an emulsifier mixture comprised of Span 80:Brij 35 (25%). Nevertheless, extensive study, such as focusing on interfacial tension and interfacial dilational rheology properties, is recommended in order to provide a more comprehensive understanding on the interface formed by Span 80:Brij 35 and their effect on emulsion stability.
